# MiR-142 modulates human pancreatic cancer proliferation and invasion by targeting hypoxia-inducible factor 1 (HIF-1α) in the tumor microenvironments

**DOI:** 10.1242/bio.021774

**Published:** 2017-01-09

**Authors:** Yebin Lu, Niandong Ji, Wei Wei, Weijia Sun, Xuejun Gong, Xitao Wang

**Affiliations:** Department of Gerneral Surgery, Xiangya Hospital, Central South University, Changsha, Hunan Province, People's Republic of China

**Keywords:** MiR-142, Pancreatic cancer, Cell proliferation, Cell invasion, HIF-1α, Hypoxia microenvironment

## Abstract

MicroRNAs regulate most protein-coding genes, including genes important in cancer and other diseases. In this study, we demonstrated that the expression of miR-142 could be significantly suppressed in pancreatic cancer specimens and cell lines compared to their adjacent tissues and normal pancreatic cells. Growth and invasion of PANC-1 and SW1990 cells were attenuated by overexpression of miR-142 *in vitro*. With the help of bioinformatics analysis, hypoxia-inducible factor 1 (HIF-1α) was identified to be a direct target of miR-142, and a luciferase reporter experiment confirmed this discovery. Overexpression of miR-142 decreases protein expression of HIF-1α. In the hypoxic microenvironment, HIF-1α was up-regulated while miR-142 was down-regulated. The invaded cells significantly increased in the hypoxic microenvironment compared to the normoxic microenvironment. The hypoxia treatment induced cells’ proliferation, and invasion could be inhibited by miR-142 overexpression or HIF-1α inhibition. Moreover, expression of epithelial-mesenchymal transition (EMT) markers, Vimentin, VEGF-C and E-cad, was altered under hypoxia conditions and regulated by miR-142/HIF-1α. Above all, these findings provided insights on the functional mechanism of miR-142, suggesting that the miR-142/HIF-1α axis may interfere with the proliferative and invasive properties of pancreatic cancer cells, and indicated that miR-142 could be a potential therapeutic target for pancreatic cancer.

## Abstract (precis)

**Summary:** Our findings provide insights to the functional mechanism of miR-142, suggesting that the miR-142/HIF-1α axis may interfere with the proliferative and invasive property of pancreatic cancer cells.

## INTRODUCTION

Pancreatic cancer is regarded as highly malignant, known for its progressive growth, early-stage metastasis, and poor response to both radiotherapy and chemotherapy. In the past 10 years, despite newly-approved therapeutic methods and large evolutions regarding nursing care, no revolutionary progress has been seen in the survival rate of pancreatic cancer patients (Kozak et al., 2015b). Therefore, exploring specific biomarkers which will enable earlier diagnosis and support personalized managements for treating patients at high risk of pancreatic cancer has become more and more urgent (Kozak et al., 2015a).

In almost all malignancy studies, microRNA (miRNAs) are found especially critical because they can be the results of chromosome lesions, modulated by classic cell signaling, and they themselves can act as both oncogenes and tumor suppressors (Bartel, 2004; Lan et al., 2015). Recently, a close association between miRNAs and pancreatic cancer tumorigenesis has been elucidated (Lai et al., 2015; Pavlakis et al., 2013). Various miRNAs, such as miR-200 (Yu et al., 2010), miR-146a (Li et al., 2010), miR-486 (Mees et al., 2009) and the let-7 family (Li et al., 2009b) have been confirmed to be tumor suppressors; meanwhile, pancreatic cancer malignancy was found positively associated with miR-196a (Huang et al., 2014), miR-212 (Ma et al., 2014) and miR-31 (Laurila et al., 2012). We chose to focus on miR-142 because it is closely related to many different malignancies, such as osteosarcoma (Zheng et al., 2015), colorectal carcinoma (Ghanbari et al., 2015) and liver cancer (Zhang et al., 2014). These findings suggest that miR-142 could play a tumor-inhibitive role in the above cancers; however, as far as we know, its potential functional mechanisms remain uncertain and need further exploration in pancreatic cancer.

Hypoxia-inducible factor 1α (HIF-1α), the oxygen-regulated subunit of heterodimeric basic-helix-loop-helix-PER-ARNT-SIM (bHLH-PAS) transcription factor hypoxia-inducible factor 1 (HIF-1), could regulate the transcription of genes involved in oxygen homeostasis in response to hypoxia (Tan et al., 2016; Tang et al., 2011). Hypoxia is a common feature of solid tumors in the middle- and late-stages. Hypoxia will stimulate tumor angiogenesis, increase drug resistance and enhance the migration ability (Mikhaylova et al., 2008; Tan et al., 2015). HIF-1α plays a central role in these processes, and thus becomes the potential anticancer target of concern; however, whether HIF-1α is involved in the regulation of pancreatic cancer proliferation and invasion under hypoxia microenvironment still remained unclear.

In this research, it was confirmed that miR-142, known as tumor-suppressive miRNA, had a regulatory relationship with an oncogene, HIF-1α. We confirmed that miR-142 inhibited pancreatic cancer cell proliferation and invasion, partly by choosing HIF-1α as its binding site. The HIF-1α level in cells was downregulated by manual overexpression of miR-142, which also brought down the protein expression of vimentin and VEGF-C while promoting the protein expression of E-cad under hypoxic microenvironments, suggesting that miR-142 could inhibit the hypoxic microenvironment-induced epithelial-mesenchymal transition (EMT). These findings bring about novel knowledge of miR-142's character and functional mechanism regarding the pathobiology of pancreatic cancer. Besides, a potential therapeutic blueprint for treating pancreatic cancer might be developed accordingly in the future.

## RESULTS

### miR-142 is largely downregulated in both pancreatic cancer tissues and cell lines and hinders the proliferation and invasion of pancreatic cancer cells

**Fig. 1.**
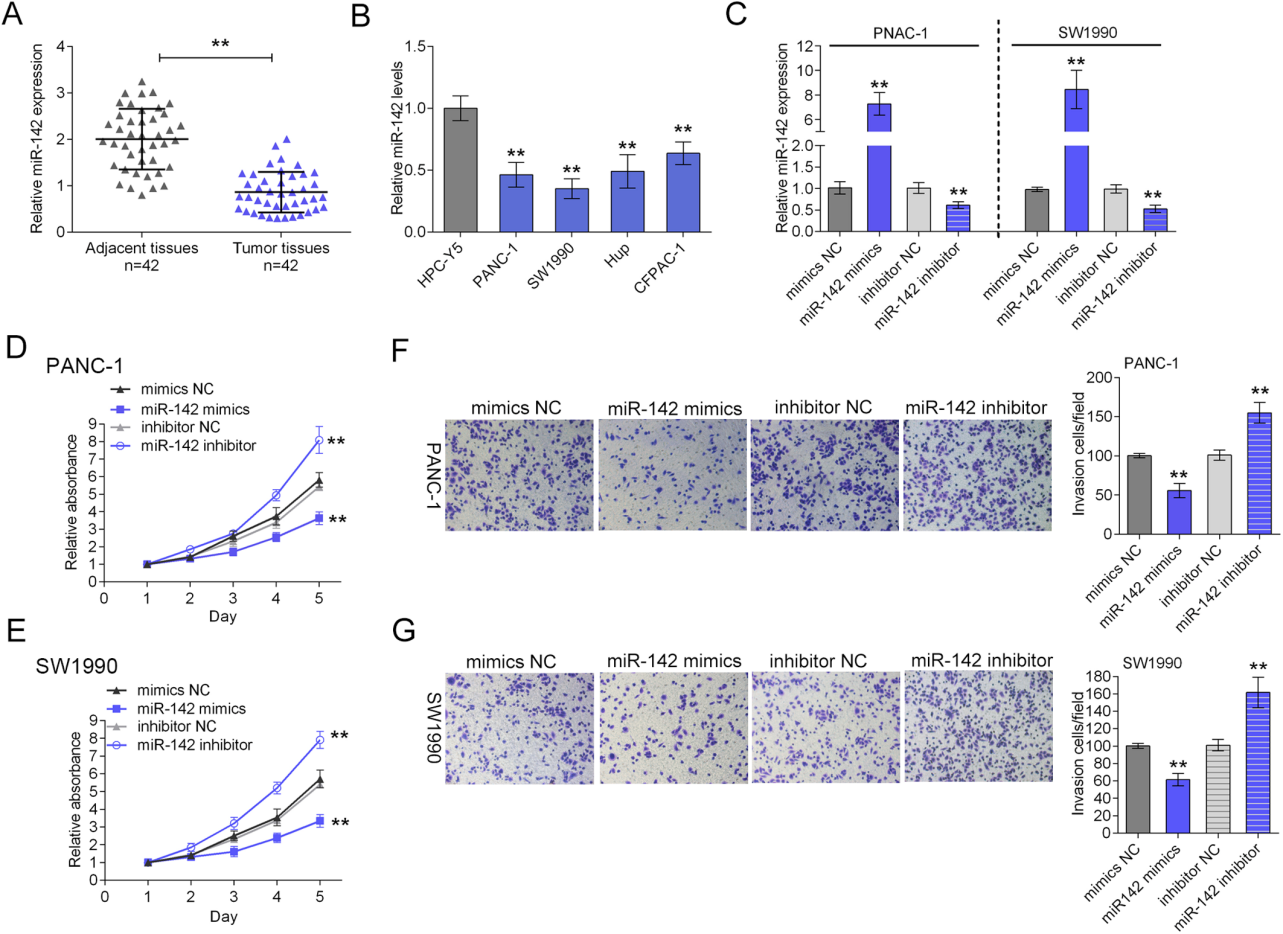
**miR-142 is largely downregulated in both pancreatic cancer tissues and cell lines and hinders the proliferation and invasion of PANC-1 and SW1990 cells.** (A) The expression of miR-142 was determined using SYBR green quantitative PCR in pancreatic cancer tissues compared to the adjacent normal tissues in a panel of matched tissues from 42 pancreatic cancer patients. (B) The miR-142 expression was determined in four pancreatic cancer cell lines compared with human pancreas cell line, HPC-Y5 using real-time PCR. (C) PANC-1 and SW1990 cell line were transfected with miR-142 mimics or miR-142 inhibitor, respectively, and the transfection efficiency was verified using real-time PCR. (D,E) The proliferation of PANC-1 and SW1990 cells in response to miR-142 overexpression or inhibition was determined using MTT assay. (F,G) The invasion of PANC-1 and SW1990 cells in response to miR-142 overexpression or inhibition was determined using Transwell assay. **P<0.01; one-way ANOVA. The data are showed as mean±s.d. of three independent experiments.

To quantify the expressional level of miR-142 in pancreatic cancer tissues and cell lines, SYBR green quantitative PCR analysis was performed. Among a large group of 42 case pairs of primary pancreatic cancer tissues and their adjacent normal tissues, a significantly lower level of miR-142 was observed in pancreatic cancer tissues compared with the paired adjacent normal tissues (Fig. 1A). Besides, compared with normal cell line HPC-Y5, the miR-142 expression level was significantly lower in all four human pancreatic cancer cell lines, PANC-1, SW1990, Hup and CFPAC-1 (Fig. 1B).

Since the expression was lower in PANC-1 and SW1990 cells, the two cell lines were chosen for further experiments. The function of miR-142 in pancreatic cancer cell lines was then investigated. PANC-1 and SW1990 cell lines were transfected with miR-142 mimics or miR-142 inhibitor to achieve miR-142 overexpression or inhibition, respectively, and the transfection efficiency was verified using real-time PCR (Fig. 1C). MTT and Transwell assays were performed to monitor the cancer cell proliferation and invasion. Results from MTT assays revealed that over-expression of miR-142 inhibited the replication of the PANC-1 and SW1990 cell lines while down-regulation of miR-142 promoted the proliferation of the PANC-1 and SW1990 cell lines (Fig. 1D,E). Moreover, Transwell assays (Fig. 1F,G) showed that cell invasion ability was significantly inhibited by ectopic expression of miR-142 in PANC-1 and SW1990 cells. By comparison, when the miR-142 inhibitor silenced endogenous miR-142, the cell invasion (Fig. 1F,G) was enhanced. These results indicate that miR-142 appeared to be tumor-suppressive in pancreatic cancer cell lines *in vitro*.

### miR-142 inhibits the expression of HIF-1α via directly binding to HIF-1α 3′ UTR

**Fig. 2.**
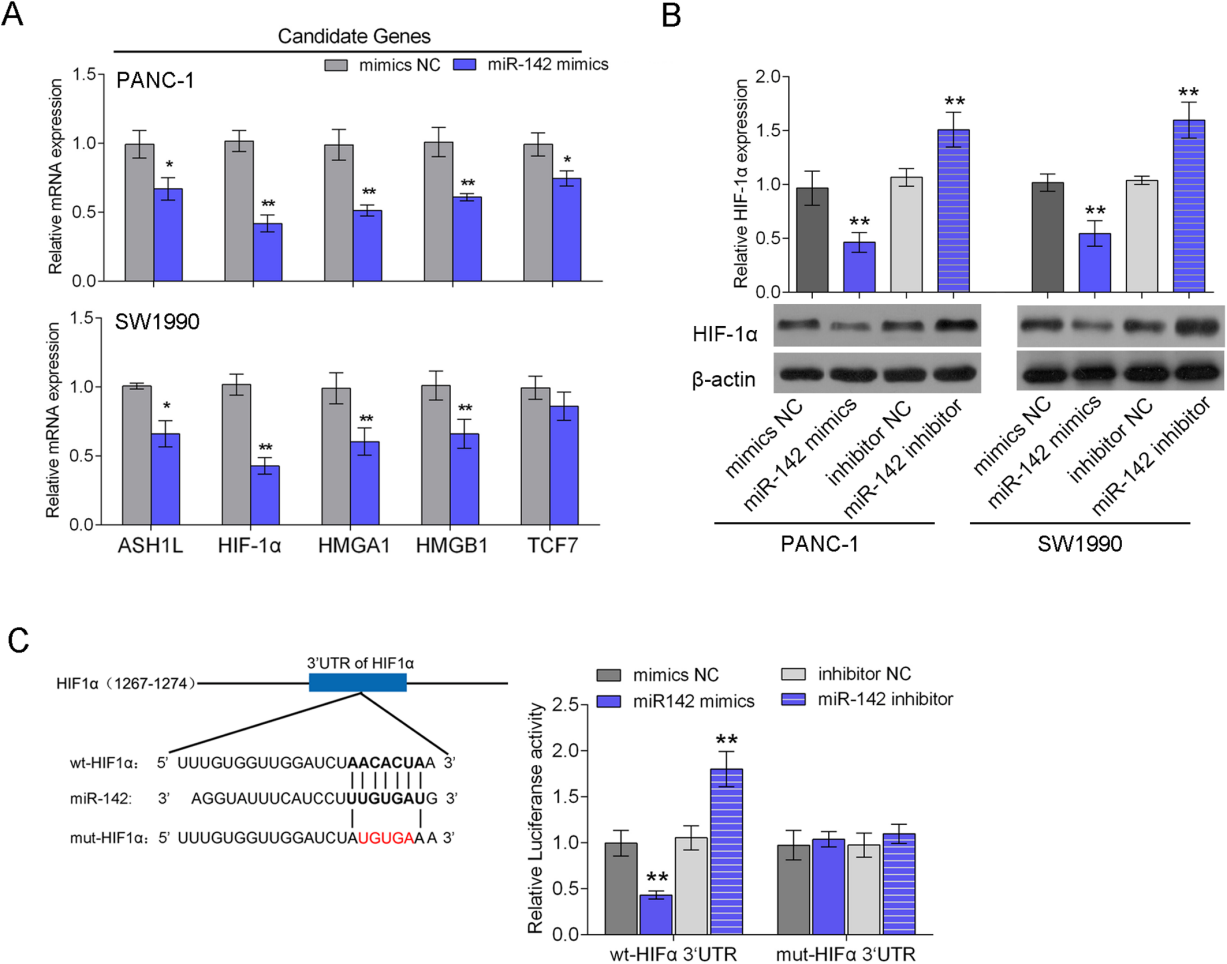
**miR-142 inhibits the expression of HIF-1α via directly binding to HIF-1α 3′ UTR.** The TargetScan, miRanda and miRWalk online tools were used to predict potential targets of miR-142. (A) The expression levels of five candidate targets, ASH-1L, HIF-1α, HMGA1, HMGB1, TCF7, were determined in response to miR-142 overexpression in PANC-1 and SW1990 cells using real-time PCR. (B) The protein expression of HIF-1α was determined in response to miR-142 overexpression or inhibition in normaxia using western blot assays. (C) The predicted miR-142 binding site within the 3′ UTR of *HIF-1α* and the mutated version generated by site mutagenesis are shown. SW1990 cells were co-transfected with wt-HIF-1α/mut-HIF-1α and miR-142 mimics or miR-142 inhibitor. The changes of the luciferase activity were monitored. *P<0.05, **P<0.01; one-way ANOVA. The data are showed as mean±s.d. of three independent experiments.

To clarify the molecular mechanism of how miR-142 inhibits the growth of pancreatic cancer cell, the TargetScan (http://www.targetscan.org/vert_71/), miRanda (http://www.microrna.org/microrna/home.do) and miRWalk (http://zmf.umm.uni-heidelberg.de/apps/zmf/mirwalk2/) online tools were used to predict potential targets of miR-142. The expression levels of five candidate targets, ASH-1L, HIF-1α, HMGA1, HMGB1, TCF7, were determined in response to miR-142 overexpression in PANC-1 and SW1990 cells (Fig. 2A). Among the monitored gene candidates, HIF-1α was chosen for further experiments because the expression of HIF-1α was reduced the most significantly (Fig. 2A). The protein expression of HIF-1α was then determined in response to miR-142 overexpression or inhibition. As exhibited by western blot assays, increased miR-142 level in PANC-1 and SW1990 cell lines significantly repressed HIF-1α protein expression, and knockdown of miR-142 increased the protein level of HIF-1α as compared with negative control (NC) (Fig. 2B). These data suggested that miR-142 inhibits the expression of HIF-1α in pancreatic cancer cells.

To further investigate the underlying mechanism, a wild-type HIF-1α 3′ UTR luciferase reporter vector (wt-HIF-1α) was created to confirm this prediction, accompanied by a mutated HIF-1α 3′ UTR luciferase reporter vector (mut-HIF-1α) by manually mutating the sequence of the putative 5 base pair miR-142 binding site in the 3′ UTR region of HIF-1α (Fig. 2C). The miR-142 mimic/inhibitor and wt-HIF-1α vector were co-transfected into the SW1990 cell line. Compared to control cells, in miR-142 mimic transfected cells the HIF-1α 3′ UTR's luciferase activity was significantly reduced, while it was increased in miR-142 inhibitor transfected cells (Fig. 2C). Besides, the miR-142-mediated repression of HIF-1α 3′ UTR luciferase reporter activity could be abolished by mutating the putative miR-142 binding sequence in the HIF-1α 3′ UTR (Fig. 2C).

### miR-142 expression is negatively correlated with HIF-1α expression in pancreatic cancer tissues

**Fig. 3.**
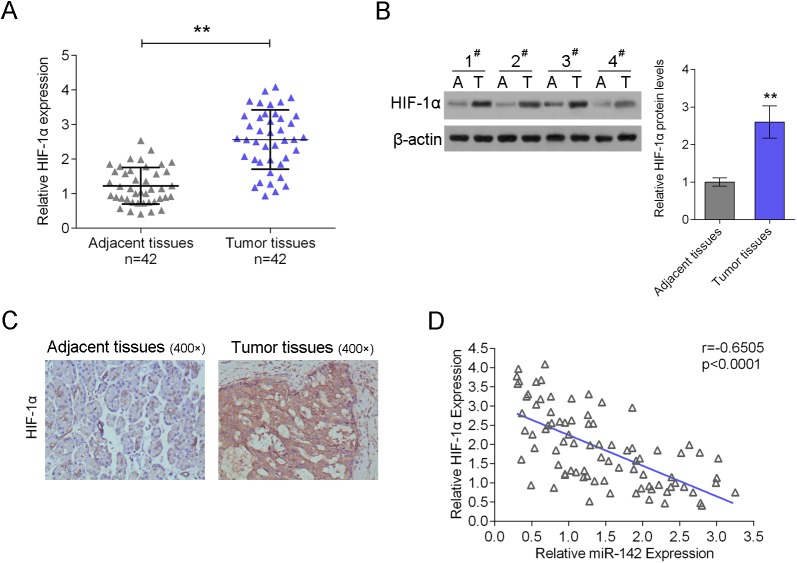
**MiR-142 expression is negatively correlated with HIF-1α expression in pancreatic cancer tissues.** (A) The expression of HIF-1α was determined using SYBR green quantitative PCR in pancreatic cancer tissues compared to the adjacent normal tissues in a panel of matched tissues from 42 pancreatic cancer patients. (B) Western blot analysis and (C) immunohistochemical analysis were used to measure the protein levels of HIF-1α in pancreatic cancer tissues compared to the adjacent normal tissues. (D) An inverse correlation between miR-142 and HIF-1α expression levels were observed. **P<0.01; one-way ANOVA and Spearman's rank correlation analysis. The data are showed as mean±s.d. of three independent experiments.

Next, SYBR green quantitative PCR analysis was performed to quantify the mRNA expressional level of HIF-1α in pancreatic cancer tissues. Results showed that HIF-1α expression increased in pancreatic cancer tissues compared with the adjacent normal tissues (Fig. 3A), the western blot analysis and immunohistochemical analysis showed similar results – that the protein expression of HIF-1α was significantly higher in pancreatic cancer tissues than adjacent normal tissues (Fig. 3B,C). An inverse correlation was observed between miR-142 and HIF-1α expression levels (Fig. 3D).

### miR-142/ HIF-1α axis regulates hypoxia-induced cell proliferation and invasion

**Fig. 4.**
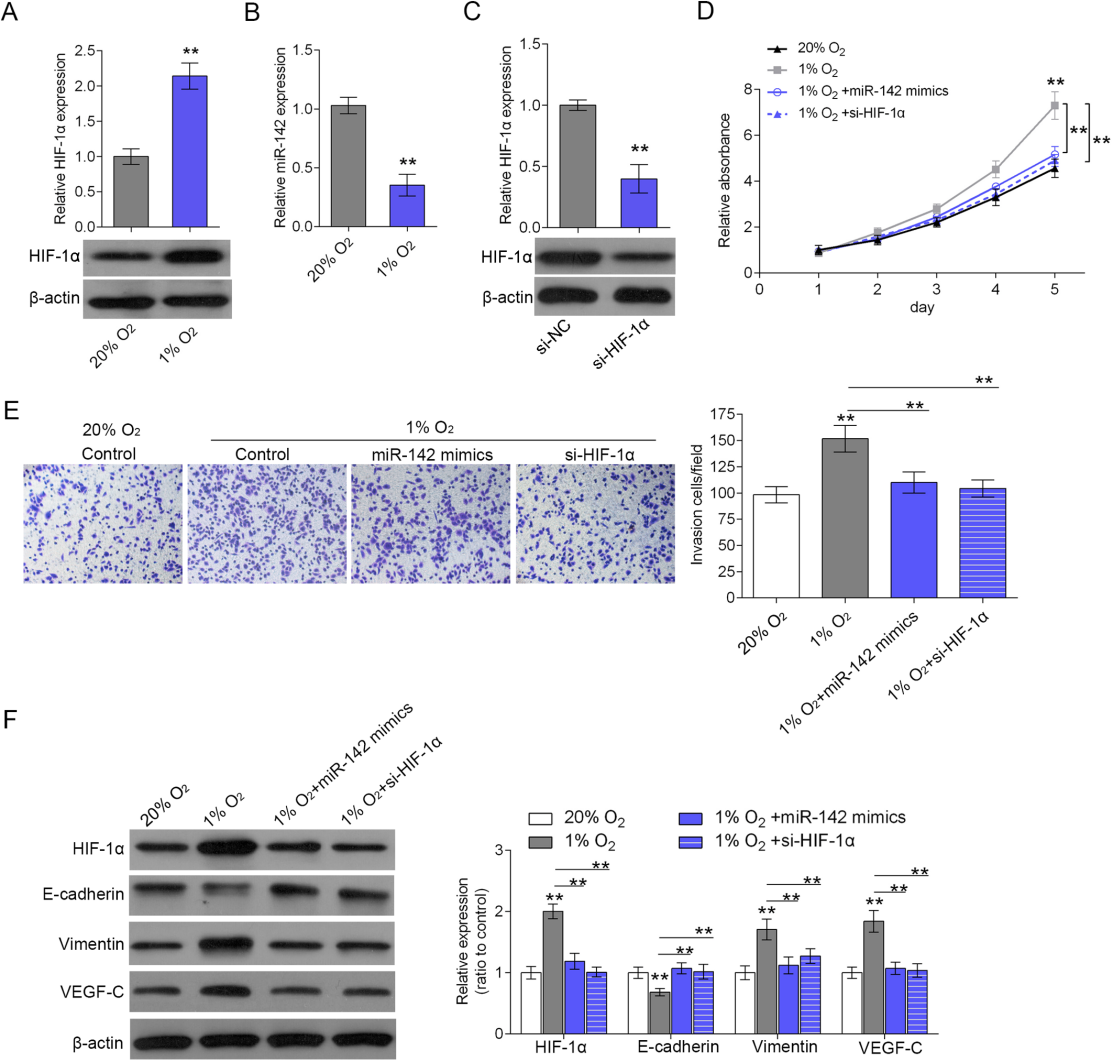
**miR-142/ HIF-1α axis regulates hypoxia-induced cell proliferation and invasion.** (A) The protein expression of HIF-1α was determined in hypoxia (1% O_2_) treatment compared to that in the normoxia (20% O_2_) condition as exhibited by western blot assays. (B) The expression of HIF-1α was determined in hypoxia (1% O_2_) treatment compared to that in the normoxia (20% O_2_) condition as exhibited by real-time PCR assays. (C) si-HIF-1α was transfected into SW1990 cells to achieve HIF-1α knockdown. The transfection efficiency was verified using western blot assays. (D) The SW1990 cells’ proliferation in response to miR-142 overexpression or HIF-1α knockdown in hypoxia (1% O_2_) treatment compared to that in the normoxia (20% O_2_) condition was monitored using MTT assays. (E) The SW1990 cells’ invasion in response to miR-142 overexpression or HIF-1α knockdown in hypoxia (1% O_2_) treatment compared to that in the normoxia (20% O_2_) condition was monitored using Transwell assays. (F) The protein expression of HIF-1α, E-cad, vimentin and VEGF-C was monitored in response to miR-142 overexpression or HIF-1α knockdown in hypoxia (1% O_2_) treatment compared to that in the normoxia (20% O_2_) condition using western blot assays. **P<0.01; one-way ANOVA. The data are showed as mean±s.d. of three independent experiments.

According to previous studies, hypoxia has a close association with cancer cell invasion (Chouaib et al., 2012; Raja et al., 2014; Tan et al., 2015; Tsujinaka et al., 2011). Given the key role of HIF-1α in the regulation of genes associated with angiogenesis, tumor resistance, invasion and metastasis under hypoxia conditions (Kim et al., 2015; Li et al., 2009a; Park et al., 2010), further experiments were arranged to investigate how miR-142/HIF-1α axis regulates tumor biological function in hypoxic microenvironment in pancreatic cancer. The protein expression of HIF-1α in SW1990 cells was significantly up-regulated in hypoxia (1% O_2_) treatment compared to that in the normoxia (20% O_2_) condition, as exhibited by western blot assays (Fig. 4A). Inversely, the expression of miR-142 was repressed in hypoxia (1% O_2_) treatment compared to that in the normoxia (20% O_2_) condition, as exhibited by real-time PCR assays (Fig. 4B). si-HIF-1α was transfected into SW1990 cells to achieve HIF-1α knockdown, and the inhibitory efficiency was verified using western blot assays (Fig. 4C). The proliferation and invasion of SW1990 cells in response to miR-142 overexpression or HIF-1α knockdown in hypoxia (1% O_2_) treatment or normoxia (20% O_2_) condition was then monitored using MTT and Transwell assays (Fig. 4D,E). Results showed that hypoxia could significantly promote SW1990 cell proliferation and invasion compared with that of the normoxia condition, while the promotive effect of hypoxia on SW1990 cell proliferation and invasion could be partly restored by miR-142 overexpression and HIF-1α knockdown (Fig. 4D,E). As exhibited by the western blot assays, the protein expression of HIF-1α, vimentin and VEGF-C was up-regulated, while the protein expression of E-cad was down-regulated by hypoxia, suggesting that EMT transformation was induced in the hypoxic microenvironment (Fig. 4F). After either HIF-1α knockdown or miR-142 overexpression, the effect of hypoxia on the above factors could be partly restored, thus regulating the cell proliferation and invasion (Fig. 4F). Taken together, miR-142/HIF-1α axis regulates hypoxia-induced cell proliferation and invasion of pancreatic cancer.

### The correlations between miR-142/HIF-1α axis and the pathological stage of pancreatic cancer

To validate the correlation between miR-142/HIF-1α axis and the pathological stage in pancreatic cancer, 42 pancreatic cancer cases were analyzed, and the relative expression levels of HIF-1α and miR-142 according to clinicopathological parameters were exhibited in Table 1. As exhibited, the expression of HIF-1α (*P*=0.016) and miR-142 (*P*=0.038) was positively or negatively correlated with the stage of pancreatic cancer, respectively, and miR-142 was negatively correlated with lymphatic metastasis (*P*=0.043). Taken together, miR-142/HIF-1α axis was correlated with the pathological stage of pancreatic cancer, and miR-142 was correlated with lymphatic metastasis of pancreatic cancer.

**Table 1.**
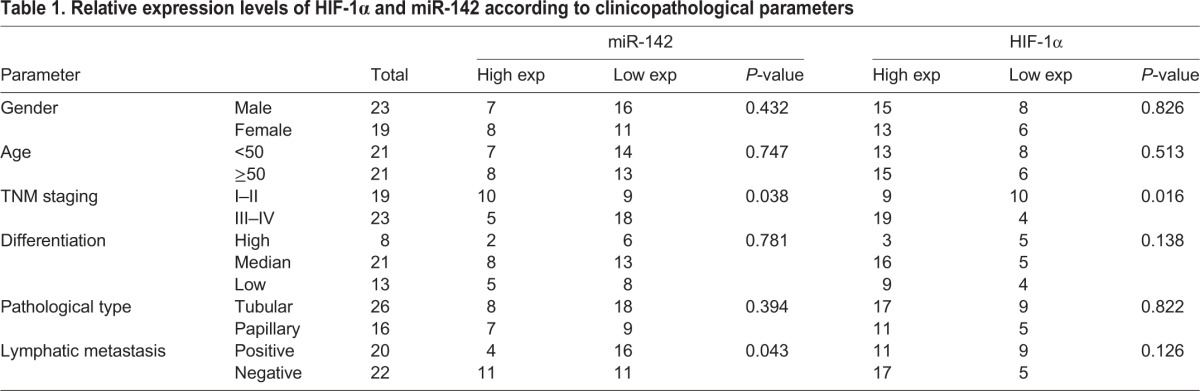
**Relative expression levels of HIF-1α and miR-142 according to clinicopathological parameters**

## DISCUSSION

Abnormalities in miRNAs’ expression are observed almost in all fields of malignant biology, such as cell replication, apoptosis, invasion and/or metastasis, and they may serve as either tumor inhibitors or promoters (Hwang and Mendell, 2007). In this study, we chose miR-142 as the research aim because of its potential suppressive function in human malignancies. Song et al. (2009) indicated that cell proliferation in both colorectal carcinoma and osteogenic sarcoma cell lines was inhibited by overexpression of miR-142. Nevertheless, so far, miR-142's role in pancreatic cancer tumorigenesis and the detailed functional mechanisms of how miR-142 performs remain uncertain. In our study, the real-time PCR results indicated that miR-142's expressional level was obviously down-regulated both in pancreatic cancer tissues and cell lines. Manual enhanced expression of miR-142 significantly inhibited the proliferation and invasion of pancreatic cancer cells. Conversely, knocking down of miR-142 might accelerate cell growth and invasion. The above results expressed that miR-142 could potentially be tumor-suppressive in pancreatic cancer.

Since the effect of certain miRNAs on carcinogenesis depends on their downstream targets (Chen et al., 2015; Profumo et al., 2015), TargetScan, miRanda and miRWalk online tools were used to predict the candidate targets of miR-142. Among five candidates, HIF-1α was shown to be the most significantly down-regulated by miR-142 overexpression. Overexpression of miR-142 could down-regulate the protein level of HIF-1α, while knockdown of miR-142 could up-regulate the expression of HIF-1α. The luciferase reporter assay confirmed the inhibitive effect on the translation of miR-142's putative binding in the HIF-1α 3′UTR region in SW1990 cells. Moreover, in surgical pancreatic cancer samples, the expressional level of HIF-1α significantly increased, and inversely correlated with miR-142 expression.

According to previous studies, hypoxia has a close association with tumor angiogenesis and cancer cell invasion (Chouaib et al., 2012; Raja et al., 2014; Tan et al., 2015; Tsujinaka et al., 2011). As a result of deprivation of oxygen (hypoxia), the growth and viability of cells is reduced. HIF-1α helps to restore oxygen homeostasis by inducing glycolysis, erythropoiesis and angiogenesis. HIF-1α has been reported to play a key role in the regulation of genes associated with angiogenesis, tumor resistance, invasion and metastasis under hypoxia conditions (Kim et al., 2015; Li et al., 2009a; Park et al., 2010). To further confirm whether the miR-142/HIF-1α axis was involved in the regulation of pancreatic cancer cell proliferation and invasion under hypoxia treatment, the cell proliferation and invasion was monitored under hypoxia conditions, as well as the expression alternation of invasion-related factors. As expected, the protein expression of HIF-1α, vimentin and VEGF-C was up-regulated, while the protein expression of E-cad was down-regulated by hypoxia, suggesting that EMT transformation was induced in a hypoxic microenvironment. Moreover, the promotive effect of hypoxia on cancer cell proliferation and invasion could be partly restored after either miR-142 overexpression or HIF-1α knockdown. In addition, 42 pancreatic cancer cases were analyzed, and the expression of HIF-1α and miR-142 was correlated with the stage of pancreatic cancer, and miR-142 was correlated with lymphatic metastasis.

In summary, it was identified that through targeting HIF-1α, pancreatic cancer cell proliferation and invasion are inhibited by miR-142; miR-142/ HIF-1α axis regulates hypoxia-induced cell proliferation and invasion through regulation of E-cad, vimentin and VEGF-C. The miR-142/HIF-1α axis established in the present study may be essential in modulating pancreatic cancer cell growth and invasion, and may serve as a new medical portal for pancreatic cancer treatment in the future.

## MATERIALS AND METHODS

### Tissue samples, cell lines and cell transfection

This project was approved by Xiangya Hospital of Central South University Committee on Biomedical Research Ethics. All patients signed informed consent. This project was conducted according to the Declaration of Helsinki Principles. This project was approved by the Ethics Committee of Xiangya Hospital of Central South University. We collected 42 pairs of surgical tissue samples of pancreatic cancer, each including the adjacent normal tissues. All samples are obtained from Surgical Department at Xiangya Hospital of Central South University (Changsha, China). Once resected, the tissues were instantly frozen by soaking in liquid nitrogen for further RNA and protein extraction or fixed in 4% paraformaldehyde for immunohistochemical analysis.

We purchased four human pancreatic cancer cell lines, including PANC-1, SW1990, Hup, CFPAC-1 and a normal cell line HPC-Y5 from the American Type Culture Collection (ATCC, Manassas, VA, USA). All cells were cultured in RPMI-1640 medium (Invitrogen, USA) containing 10% fetal bovine serum (GIBCO, USA) at 37°C in a humidified atmosphere with 5% CO_2_. Intervention of miR-142 expression was conducted by transfection with miR-142 mimics or miR-142 inhibitor (Genepharma, Shanghai, China) with the help of Lipofectamine 2000 (Invitrogen). si-HIF-1α was used to achieve knockdown of HIF-1α (GeneCopoecia, Guangzhou, China). Cells were seeded in 96-well plates or 6-well plates, transfected, and incubated for 48 h before RNA/protein extraction.

### Immunohistochemical analysis

As previously described (Lu et al., 2015), the tissue sections were dewaxed and washed three times with PBS. For non-specific inhibition, each section was incubated in 10% normal goat serum (Boster, Wuhan, China) for 30 min at 37°C and then with primary antibody, HIF-1α (Abcam, USA), overnight at 4°C. The sections were washed three times with PBS and were incubated with secondary antibody linked with biotin, and then with HRP-marked anti-biotin (Boster) for 30 min at 37°C. Subsequently, the sections were incubated in freshly prepared diaminobenzidine (Boster) and subsequently counterstained with hematoxylin (Boster). The sections were then observed under an optical microscope (200×) (Olympus, Tokyo, Japan).

### RNA extraction and SYBR green quantitative PCR analysis

Trizol reagent (Invitrogen) was used to extract total RNA from cells or tissue samples. Hairpin-it TM miRNAs qPCR kit (Genepharma, Shanghai, China) was used to detected the mature miR-142 expressional levels in cells. Expression of RNU6B served as an endogenous control. SYBR green qPCR assay (Takara, Dalian, China) helped with the measurement and calculation of expressional level. The 2^−ΔΔCT^ method was applied to process the data.

### MTT assay

A modified MTT assay was used to evaluate cell viability. Cells were transfected with the intended agent then seeded into the 96-well culture plates at a density of 2×10^3^ cells/well. Cell viability of PANC-1 and SW1990 was then assessed at five different time points (1, 2, 3, 4, 5 days). Theoretically, the enzymatic change of 3-(4,5-dimethyldiazol-2-yl)-2,5-diphenyltetrazolium bromide (MTT; Sigma-Aldrich, USA), which results in a colored formazan product, was seen as the indicator of mitochondrial dehydrogenase activity. MTT (10 μl, 10 mg/ml) was applied to the cells. After 4 h of incubation, the supernatant was removed so that the reaction could be stopped. Then, to dissolve the formazan product, 100 μl DMSO was applied to each well. 0.5 h later, the optical density (OD) at 490 nm of each well was detected with a plate reader (ELx808 Bio-Tek Instruments, Hercules, USA).

### Transwell assay

Evaluation of the invasive and migratory potential of cells was performed with Transwell inserts with 8 μm pores (Corning, New York, USA). For invasion assay, each upper insert was seeded with 3×10^5^ cells suspended in serum-free medium at 24 h after transfection. The upper inserts were pre-coated with matrigel matrix (BD, New York, USA). The lower chambers were each filled with 500 μl medium containing 10% FBS. 48 h later, we used a cotton swab to remove non-invaded cells from the upper surface of the Transwell membrane. Then, on the lower membrane surface, the invaded cells were fixed by methanol, stained with 0.1% crystal violet, photographed and counted under a microscope. For migration assay, similar procedures were conducted, except that 2×10^5^ cells were applied and the inserts were not pre-coated by matrix gel. Six random fields were counted at 100× magnification for each insert. This experiment was conducted three times separately.

### Western blot analysis

The protein expression of HIF-1α, E-cadherin, vimentin and VEGF-C in pancreatic cancer cells was detected by immunoblotting. RIPA buffer containing 1% PMSF was used to lyse the cells. Proteins were then loaded onto a 15% SDS-PAGE minigel and transferred onto PVDF membrane under an electric field. Then the blots were probed with 1:1000 diluted rabbit polyclonal antibodies against HIF-1α, E-cadherin, vimentin and VEGF-C (Abcam, USA) at 4°C overnight. After that, we incubated the blots with HRP-conjugated secondary antibody (1:5000). ECL substrates were used to visualize signals (Millipore, Billerica, USA). For normalization, the endogenous β-actin was also tested.

### Luciferase reporter assay

After seeding into a 24-well plate, SW1990 cells were cultured overnight, then co-transfected with the wild-type/mutated HIF-1α 3′ UTR luciferase reporter vector and pRL-TK plasmids, or transfected with miR-142 mimics/miR-142 inhibitor. 48 h after transfection, the luciferase assays were performed with the help of the Dual Luciferase Reporter Assay System (Promega, Madison, USA).

### Hypoxia treatment

SW1990 cells or miR-142 mimics or si-HIF1-α transfected cells were seeded into 6-well plates or Transwell upper inserts, cultured in hypoxia chamber in a humidified atmosphere with 94% N_2_, 5% CO_2_ and 1% O_2_ for 24 h then change to normoxia for additional 24 h for real-time PCR, western blot analysis and Transwell assay. For MTT assay, cells seeded in 96-well plates and cultured in hypoxia condition for 24 h then change to normoxia for additional 24, 48, 72 and 96 h.

### Statistical analysis

Data were processed using SPSS 17.0 statistical software (SPSS, Chicago, IL, USA), and displayed as mean±s.d. of three independent experiments. By using Wilcoxon's paired test, we compared the expression of miR-142 in pancreatic cancer surgical biopsy tissues and the adjacent normal tissues of them. One-way ANOVA was used to evaluate the differences between groups in terms of cell proliferation and invasion. *P* values of <0.05 were considered to be of statistical significance.
