# Correction: MiR-142 modulates human pancreatic cancer proliferation and invasion by targeting hypoxia-inducible factor 1 (HIF-1α) in the tumor microenvironments

**DOI:** 10.1242/bio.059951

**Published:** 2023-06-19

**Authors:** Yebin Lu, Niandong Ji, Wei Wei, Weijia Sun, Xuejun Gong, Xitao Wang

There was an error published in *Biol. Open* (2017) **6**, 252-259 (doi:10.1242/bio.021774).

The image from the si-HIF-1α group in Fig 4E was inadvertently duplicated in the mimics NC group in Fig 1G. Replicate SW1990 panels have been substituted. The corrected Fig. 1 is shown below.

**Fig. 1.**
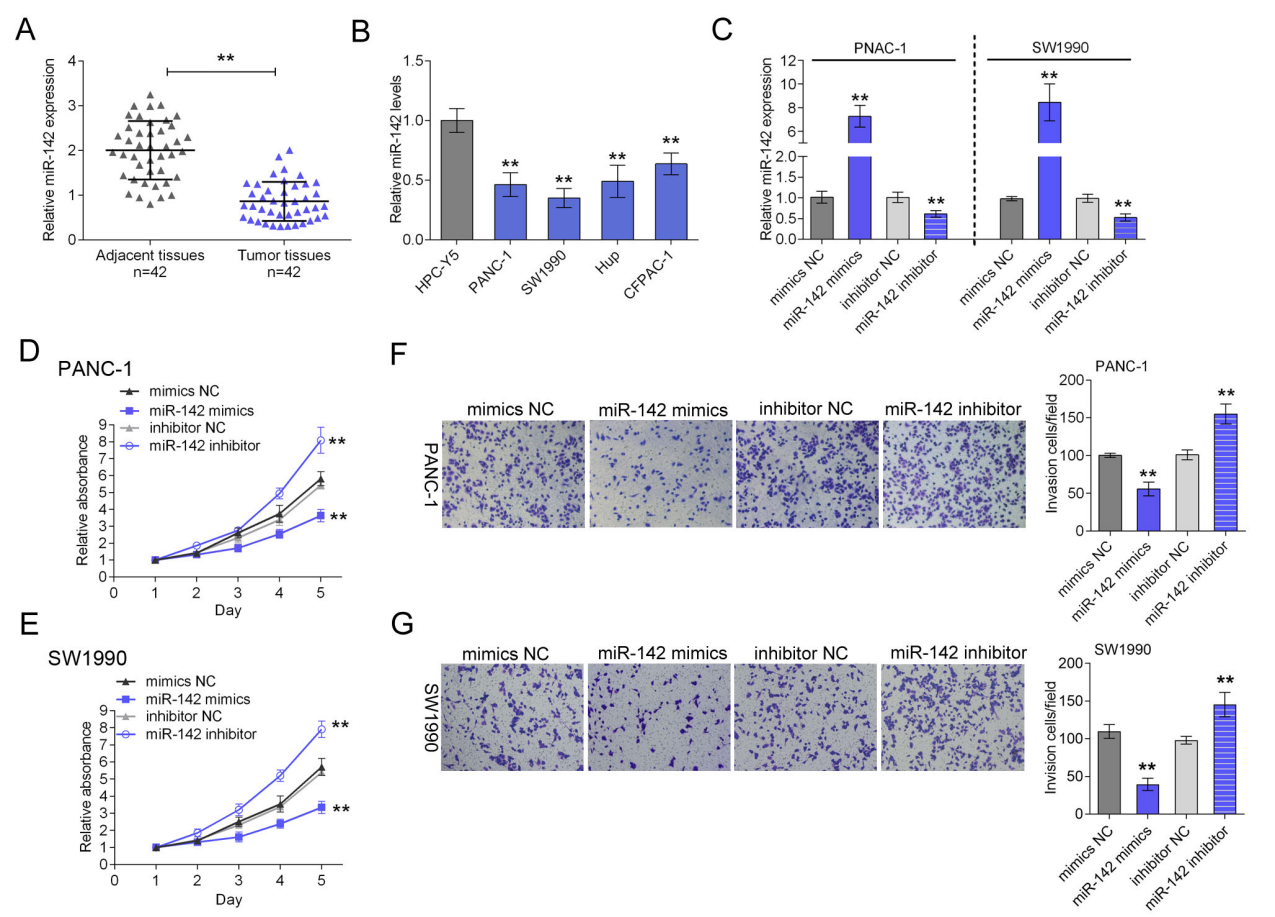
**miR-142 is largely downregulated in both pancreatic cancer tissues and cell lines and hinders the proliferation and invasion of PANC-1 and SW1990 cells.** (A) The expression of miR-142 was determined using SYBR green quantitative PCR in pancreatic cancer tissues compared to the adjacent normal tissues in a panel of matched tissues from 42 pancreatic cancer patients. (B) The miR-142 expression was determined in four pancreatic cancer cell lines compared with human pancreas cell line, HPC-Y5 using real-time PCR. (C) PANC-1 and SW1990 cell line were transfected with miR-142 mimics or miR-142 inhibitor, respectively, and the transfection efficiency was verified using real-time PCR. (D,E) The proliferation of PANC-1 and SW1990 cells in response to miR-142 overexpression or inhibition was determined using MTT assay. (F,G) The invasion of PANC-1 and SW1990 cells in response to miR-142 overexpression or inhibition was determined using Transwell assay. **P<0.01; one-way ANOVA. The data are showed as mean±s.d. of three independent experiments.

In addition, there is a possible duplication of the mimics NC and inhibitor NC HIF-1α blots in Fig 2B. The authors no longer have the original data for these blots, but state that new data from repeated experiments (not shown) is consistent with the results reported in the paper. The additional data are available from the authors upon reasonable request.

The authors apologise to readers for these errors, which do not impact the results or conclusions of this paper.

